# Acute bolus obstruction following surgical treatment of paraesophageal herniation of the greater omentum

**DOI:** 10.1093/jscr/rjab208

**Published:** 2021-05-27

**Authors:** S Rassam, T Steffen, P Folie

**Affiliations:** Department of Surgery, Hospital of the Canton of St. Gallen, St. Gallen, Switzerland; Department of Surgery, Hospital of the Canton of St. Gallen, St. Gallen, Switzerland; Department of Surgery, Hospital of the Canton of St. Gallen, St. Gallen, Switzerland

## Abstract

Hiatal herniations are most commonly diagnosed during work-up for gastroesophageal reflux disease. Here, we present a patient with retrosternal pain for whom the computed tomography scan showed a lipomatous formation in the lower posterior mediastinum, and further examination indicated the origin to be paraesophageal herniation (PEH) of the greater omentum. This was confirmed by laparoscopy, the herniated part of the greater omentum was repositioned and the hiatal hernia was repaired. During recovery the patient complained of dysphagia, a common and transient postoperative occurrence, but which later proved to be a mechanical obstruction caused by a bolus. This case raises awareness of potential differential diagnoses pre- and postoperatively in conjunction with PEH, and the management of such differential diagnoses is discussed.

## INTRODUCTION

Numerous complications can occur after surgical treatment of a paraesophageal hernia (PEH), such as postoperative leakage, early recurrence of the hernia and stenosis resulting from oedema or poor operative technique. In the latter situation, contrast oesophagogram and/or oesophagogastroscopy are needed to confirm diagnosis. If stenosis is caused by oedema, the therapeutic approach would be to wait and see, with optional support by oral decongestive medication. In cases without improvement, balloon dilatation via endoscopy would be performed to ameliorate passage. In situations where postoperative clinical deterioration is caused by tight hiatoplasty or oesophageal or vascular lesions, surgical revision is required. Intrathoracic displacement of a portion of the greater omentum is frequently seen in large type III hernias due to herniation of major parts of the stomach. Exclusive omental herniation with near-orthotopic position of the cardia is rare [1, 2]. Here, we report an uncommon complication of postoperative stenosis due to an early postoperative bolus obstruction.

## CASE REPORT

**Figure 1:**
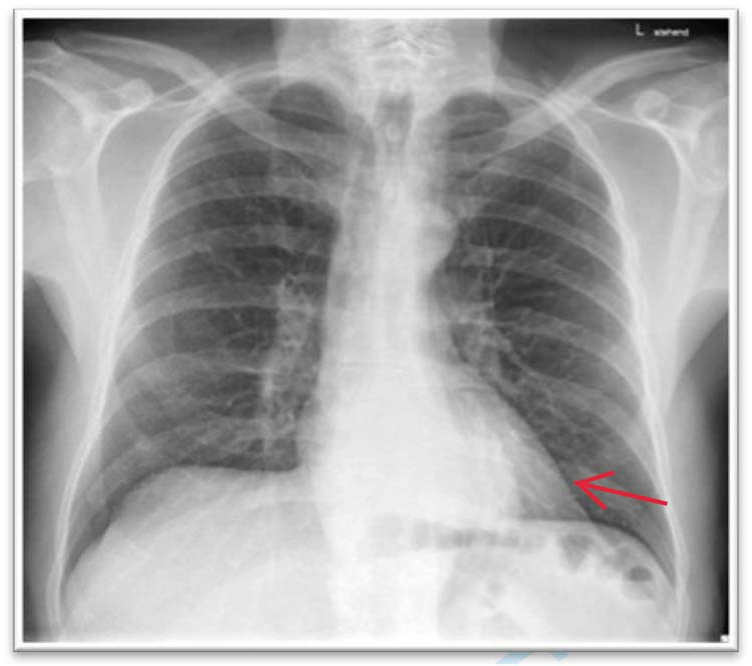
Thoracic X-ray with retrocardiac mass.

**Figure 2:**
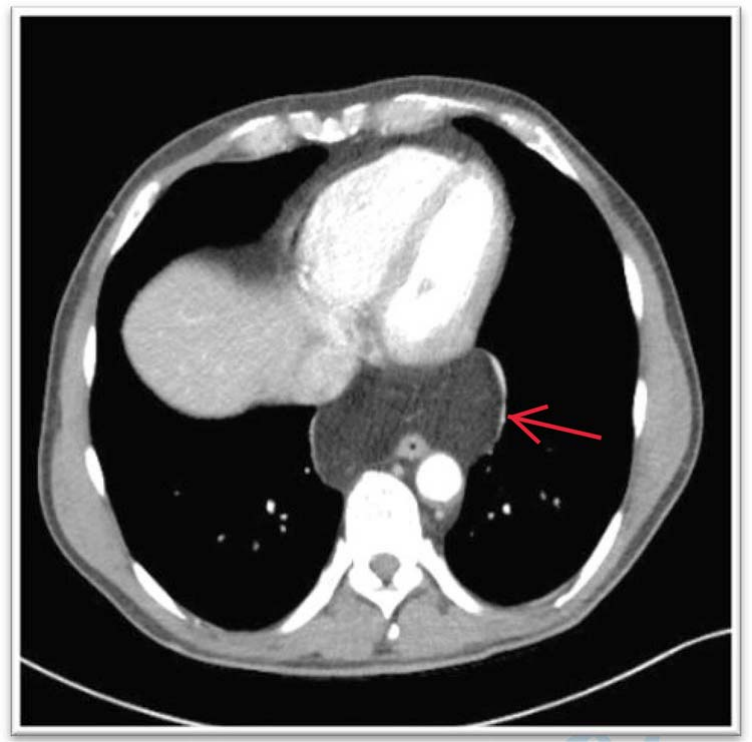
CT showing hypodense mass in the lower posterior mediastinum.

A 63-year-old male patient underwent a medical check-up due to acute bronchitis. The patient’s body mass index was 24.7 kg/m^2^, he was in good physical condition and no further pathological findings were revealed during clinical examination. Chest X-ray showed a retrocardiac mass (Fig. 1) and a subsequent CT scan indicated a lipomatous mass in the lower posterior mediastinum measuring 10 × 4.4 × 10.6 cm. A vascular pedicle reaching into the abdominal cavity suggested paraesophageal herniation of a large portion of the greater omentum (Fig. 2). The patient was referred to the surgical department suffering from a retrosternal feeling of pressure aggravated in supine position, with a differential diagnosis of lipomatous tumour. No symptoms of gastroesophageal reflux disease were reported, and the clinical examination showed no abnormalities. Preoperative work-up included a gastroscopy and contrast medium swallow. Endoscopy showed an axial hernia of 4 cm without further irregularities or signs of a reflux disease. Passage appeared physiological with no signs of gastroesophageal reflux and orthotopic positioning of the oesophagogastric junction.

**Figure 3:**
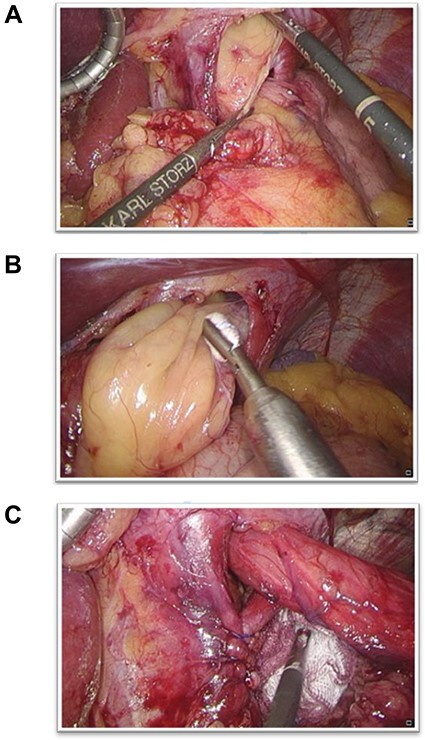
(**A**) The oesophageal hiatus with herniation of the greater omentum; (**B**) Careful withdrawal of the omentum from the oesophageal herniation; (**C**) After omental reposition, the distal oesophagus was repositioned and a dorsal crurorhaphy performed.

Given these findings, a laparoscopic exploration with omental reposition followed by hiatoplasty was scheduled. A wide oesophageal hiatus was found, with herniation of the entire greater omentum despite near-orthotopic positioning of the cardia. A 3-cm axial herniation was observed in the cardia. The greater omentum was repositioned laparoscopically. No histologic examination was performed as the lipomatous mass intraoperatively undoubtedly turned out to be a vital portion of the greater omentum just like preoperatively suggested. Hiatal dissection was performed with mobilization of the distal oesophagus as well as complete resection of the large hernial sac. After complete dissection of both diaphragmatic crus and partial detachment of the left triangular ligament, a dorsal crurorhaphy was performed (Fig. 3A–C). Because of the hernia’s large size and gaping crura, a biosynthetic mesh (Gore Bio A®, W. L. Gore & Associates, Inc., Flagstaff, Arizona, USA) was placed on the reconstructed site and secured with sutures. Lastly, a fundophrenicopexia was performed.

**Figure 4:**
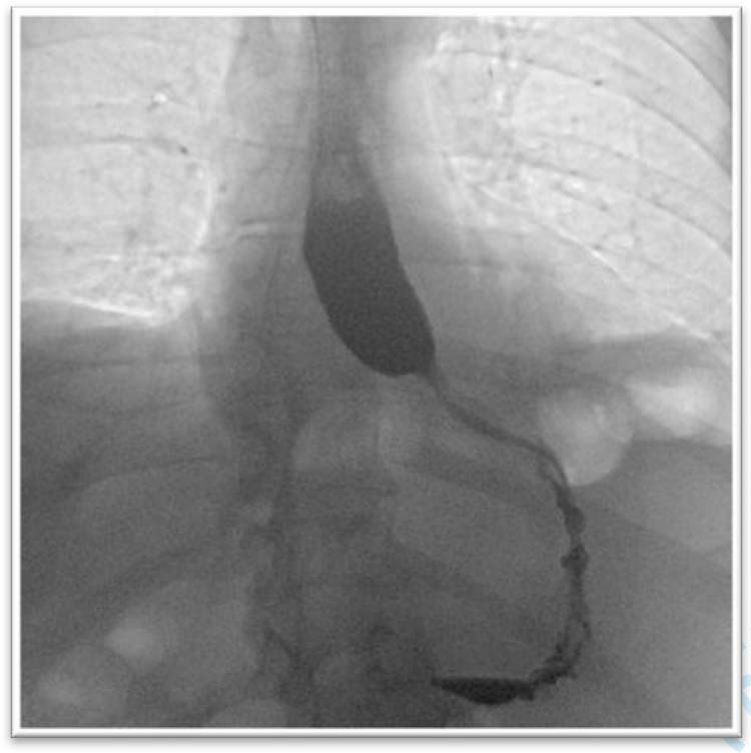
The barium oesophagogram showing a near to total stenosis caused by alimentary bolus.

We restricted the intervention to an anatomical repair of the hiatal region. Fundoplication was not indicated because preoperative examination revealed no symptoms of reflux disease. The first three postoperative days were uneventful, and the patient responded well to puréed food. After receiving solid food on postoperative day four, the patient was regurgitating multiple times per day and complaining of globus sensation. A contrast-enhanced oesophagogram showed near-complete stenosis of the esophagogastric junction (Fig. 4). Gastroscopy was crucial to avoid an unnecessary surgical exploration as it showed the stenosis was not of mechanical origins such as a too tight hiatoplasty or fundoplication but was caused by a food bolus hindering optimal passage. The bolus was removed during gastroscopy and globus sensation disappeared thereafter. The remaining postoperative period was uneventful, with normal diet and no feelings of retrosternal pressure at hospital discharge.

## DISCUSSION

Here, we present a rare but important differential diagnosis of postoperative complete obstruction of the oesophagus after surgical repair of a paraesophageal hernia. Early postoperative bolus obstruction requires endoscopic management and removal of the bolus, and unnecessary surgical revision must be prevented. Postoperative oesophagogastric endoscopy should therefore be implemented early and often.

Sole herniation of the greater omentum through the oesophageal hiatus does not fit into the existing hiatal hernia classification [3]. In the present case, a different structure than the stomach herniated with the stomach itself in a regular position. We believe this case should be classified as an atypical type IV herniation. To our knowledge laparoscopic paraesophageal omental hernia without herniation of at least part of the stomach is a rare disease, reported only a few times in the literature.

The diagnostic approach for a mediastinal mass involves detailed history-taking, physical examination, laboratory examination and imaging studies. Commonly, a chest and abdominal CT scan with intravenous contrast agent is recommended to define the herniated structures. In the present case, CT showed a lipomatous mass and a vascular pedicle reaching into the abdominal cavity which was indicative for a PEH. Radiologically visualised continuity of the herniated structure above and below the diaphragm can be a sign of either lipomatous tumour or paraesophageal omental herniation [4]. In this case, the vessel was indicative of being the gastroepiploic artery, which is the supplying vessel of the greater omentum. In some cases, only a CT-guided tissue biopsy or diagnostic laparoscopy will result in a definitive diagnosis [5, 6]. Surgical management is indicated in symptomatic patients in order to prevent complications such as intestinal obstruction, strangulation or perforation [3].

## CONCLUSION

Early postoperative bolus obstruction after surgery for hiatal hernia is a rare complication that requires endoscopic management. This is in marked contrast to the more frequent complications which require surgical intervention.

## CONFLICT OF INTEREST STATEMENT

None declared.

## FUNDING

The authors have not received grant support or research funding for this study.

## References

[1] Stephens M, Hughes A. Paraoesophageal omental hernia. Case Rep Dermatol 2013;2013:bcr2013009683. 10.1136/bcr-2013-009683 PMC3736282 23893273

[2] Yunoki J, Ohteki H, Naito K, Hisajima K. Omental herniation through the esophageal hiatus mimics mediastinal lipomatous tumor. Jpn J Thorac Cardiovasc Surg 2004;52:580–2. 15651406 10.1007/s11748-004-0028-9

[3] Dean C, Etienne D, Carpentier B, Gielecki J, Tubbs RS, Loukas M. Hiatal hernias. Surg Radiol Anat 2012;34:291–9. 22105688 10.1007/s00276-011-0904-9

[4] Kubota K, Ohara S, Yoshida S, Nonami Y, Takahashi T. Intrathoracic omental herniation through the esophageal hiatus: a case report. Radiat Med 2001;19:307–11. 11837582

[5] Lee HN, Yun SJ, Kim JI, Ryu C-W. Diagnostic outcome and safety of CT-guided core needle biopsy for mediastinal masses: a systematic review and meta-analysis. Eur Radiol 2020;30:588–99. 31418086 10.1007/s00330-019-06377-4

[6] Sueyoshi K, Inoue Y, Sumi Y, Okamoto K, Azuma D, Yoshikawa S, et al. A case of omental herniation through the esophageal hiatus successfully treated by laparoscopic surgery. Acute Med Surg 2017;4:367–70. 29123893 10.1002/ams2.288 PMC5674477

